# Population status and habitat suitability of the vulnerable common hippopotamus (*Hippopotamus amphibius*) in the Dhidhessa Wildlife Sanctuary, Southwestern Ethiopia

**DOI:** 10.1016/j.heliyon.2024.e40186

**Published:** 2024-11-08

**Authors:** Girma Gizachew Tefera, Tadesse Habtamu Tessema, Tibebu Alemu Bekere, Tariku Mekonnen Gutema

**Affiliations:** a Department of Natural Resource Management, Colleague of Agriculture and Veterinary Medicine, Jimma University, Jimma, Ethiopia; b Department of Biology, College of Computational Sciences, Jimma University, Jimma, Ethiopia

**Keywords:** Dhidhessa wildlife sanctuary, Habitat association, Habitat suitability, *Hippopotamus amphibious*, Population status

## Abstract

Common hippopotamuses (Hippopotamus amphibious) are among the top five herbivorous animals in Africa. Despite being listed as a vulnerable species by the International Union for Conservation of Nature, they are a common mammal in Ethiopia's protected areas, lakes, rivers, and marshes. However, there is insufficient data to evaluate status of the population and habitat compatibility across the most of the country. The aim of this research was to ascertain the population size of hippos and the suitability of its habitat in the Dhidhessa Wildlife Sanctuary (DWS) in southwest Ethiopia. The study was conducted between 2021 and 2022. To estimate the population size, the total count procedures were employed. The adaptability of each habitat for hippos was determined using the multi-ring buffer analysis in Arc GIS 10.2. A total of 231 and 133 hippos were observed during both the dry and wet seasons, respectively. Of the surveyed individuals, 62.08 % were adults, 20.88 % were under-adults, and 17 % were young. More hippos (45.1 %) were observed in the savanna grassland during the rainy season. Subsequently, the hippos (50.6 %) relocated to the riverine forest in the dry season. Thus, the habitats in DWS and their environs were determined to range from most suitable to unsuitable for hippos. The results showed that 58.31 % of the regions were unsuitable, 18.49 % were moderately suitable, and 23.18 % were highly suitable for hippos grazing. 7.95 % of the research area slope was suitable, 26.32 % moderate and 65.72 % not suitable and 19.8 % was considered most suitable for human interference, 46.3 % was severely disturbed, and 33.9 % was moderately affected. Based on the current investigation, it has been determined that human interference in hippos' habitats is significant. In order to protect the hippos' habitats from excessive human activities and their impacts, a buffer should be created around the DWS area.

## 1 Introduction

Until the early 20th century, the common hippopotamus (*Hippopotamus amphibious*) was once widespread in wet habitats throughout Africa from the Nile to the Cape, particularly in riverss and lakes in sub-Saharan Africa [1]. Following elephants and white rhinos, it is the third largest and heaviest terrestrial mammal [2]. Hippos are essential to the ecosystem because they create tracks in water systems, recycle nutrients by spreading highly organic waste, and provide habitat for parasites, leeches, and commensal water birds [[2], [3], [4], [5]]. However, due to loss of habitat and fragmentation, this function is losing ground in most of its historical areas. The worldwide population of hippos is estimated to be between 125,000 and 150,000, and it is decreasing at a rate of 7–20 % [[2], [6], [7]]. The main factors affecting the global hippo population are the loss and deterioration of habitats, illegal trade associated with hippopotamus products (including teeth, skulls, ivory, skin, and meat), and the increasing conflict between humans and hippos [8,9]. The IUCN Red List currently categorizes the species as "vulnerable" because of habitat fragmentation and a reduction in area over the past century [10].

Similar to many other tribes in Africa the hippo is quite common in lakes, rivers, and wetlands in most areas of Ethiopia. However, today, for the stated reasons, many suitable habitats are occupied by humans and converted into small-scale sustainable agriculture and, in some cases, into large-scale commercial agriculture [8,9,11]. Therefore, the range of this species in Ethiopia has declined considerably, and in most of the highlands, the animal is extinct locally [7]. Among the several protected areas in Ethiopia, the DWS has the greatest number of hippo population in the southwestern region [12]. In order to preserve the area for this species, the Ethiopian government declared the area to be DWS in 1970. Like most problems faced by the country's wildlife conservation areas, due to political instability and repeated drought in northern and eastern Ethiopia, the DWS is heavily encroached upon and used to alleviate war and drought-ravaged populations. In addition, nearby regions surrounding the DWS have been transformed into mechanized sugarcane plantations to supply the Arjo Dhidhessa sugar factory.

Knowledge of the local wildlife and habitat requirements of the intended species is essential for managing and preserving sensitive species such as the hippo [13]. However, these aspects are generally less well known in Ethiopia and throughout the majority of Africa [14,15]. Only a few studies have been carried out on hippos’ preservation and overall the data suggest an alarming population decline [9,12]. The new human-dominated landscape has brought about mechanized monoculture cane farming, livestock, and other economic activities that have gradually led to conflict with wildlife in the DWS, especially the relatively abundant hippo population. Consequently, it is essential to implement effective conservation strategies to guarantee the sustainable preservation of the study area, especially concerning large mammals such as the hippo population. Yet, the population status and habitat suitability of hippo have not yet been studied in the DWS. Based on this, the current research primarily aimed to assess the population size and identify appropriate habitat locations for hippos within the DWS and surrounding areas using remote sensing techniques and GIS in southwestern Ethiopia.

### 1.1 The study area

**Fig. 1:**
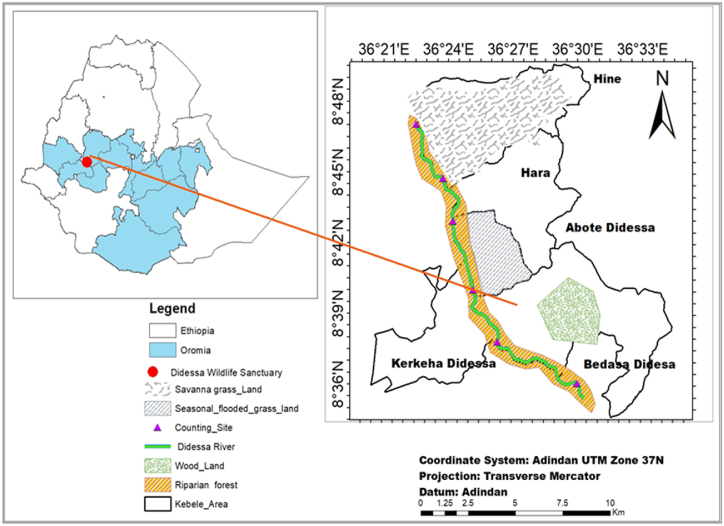
Map of study area.

The research was carried out in the DWS area and its vicinity in southwestern Ethiopia. This region is situated 395 km from Addis Ababa, positioned between 8°30′ and 8°40′ N latitude and 36°22′ and 36°43’ E longitude, covering an area of about 1300 square kilometers. It is divided into three administrative zones: East Wollega, Buno Bedele, and Jimma, all within the Oromia National Regional State (Fig. 1).

The altitudes in the DWS vary between 1350 and 1050 m above sea level. This region experiences two distinct seasons: a dry (November to February) and a wet (June to September), with precipitation levels ranging from 648 mm to 2001.8 mm. The DWS has relatively warm temperatures, with a mean annual minimum of 12 °C and a maximum of 35 °C [16]. Besides the extensive savanna and wetlands along the riverbanks, the area's terrain features moderately rugged, hilly, and mountainous landscapes. The habitats in the study area primarily consist of natural forests, wooded areas, riparian forests, seasonally flooded prairies, and broad savannah prairies, which are formed by river overflows and their permanent tributaries. This area was historically inhabited by various wildlife species, particularly antelopes, large herbivores, and carnivores. In 1970, it was assigned as DWS, mainly to protect *Hippopotamus amphibious*, a notably dominant species. Over the past decades, various factors, such as political instability and recurring droughts, have led to significant encroachment of the land prior to its legal assessment, which was utilized to settle drought-affected populations from the northern and eastern regions of the country. Meanwhile, much of the neighboring land was allocated for mechanized sugarcane farming to supply raw materials for the state-owned Arjo Dhidhessa Sugar Factory. As a result, DWS has struggled to meet its conservation goals [17].

## 2 Materials and methods

In October 2021, initial surveys were carried out to gather fundamental information about the study area. Using the collected data, appropriate research locations were selected for an in-depth examination of hippo population size and habitat suitability. Additionally, discussions took place with members of the community and factory employees to gather basic information regarding the study area. Overall, the research was completed throughout the dry season (December to February) and the rainy season (April to June). A total of six sites were selected to assess the quantity of hippos because they are close to the Dhidhessa River, which is their habitat, and are easily reachable (Table 1).

**Table 1:** Counting site of hippopotamus in Wildlife Sanctuary.

Site name	GPS location	Area/hectare	Buffer zone	Main source of water
Turee	218092.483E	1003.47	Planation farm and riverine forest	River
	955183.054N			
Kerkeha	216180.642E	286.67	Planation farm, riverine and natural forest	River
	957435.512N			
Abote	222359.872E	70	Natural forest and planation farm	River
	953199.067N			
Bedasa	216105.126E	253.35	Planation farm and riverine forest	River
	958973.479N			
Loko	215283 E	13.65	Grassland	River
	961815 N			
Sefera Tabia	213755.414E	350.75	Planation farm and riverine forest	River
	967456.88N			
Total		1977.9		

During the study period, all chosen sites were monitored for the same duration of days. Hippos were tallied based on their footprints along the riverbanks whenever pods were sighted (Table S1). For each counting occasion, a team of six observers walked along the river's edge in the DWS. Out of these six, two were documenting information on data sheets; two served as observers using binoculars, while the remaining two were armed for the team's protection against potentially dangerous wildlife. Up to 30 min were allocated for observing a group of hippos, allowing enough time to categorize individuals by age and gender. The sizes of each school and their associated river geomorphological characteristics were recorded, along with GPS coordinates. The daily average number of hippos was calculated, and monthly averages were determined. Hippos were observed twice daily, first in the morning (from 8 a.m. to 12:a.m.) and then in the afternoon (14. to 18 p.m.), as they are more visible, social, and not submerged during these times [18]. The rainy season was represented by the April and June months, given the comparatively low water levels, while December and February were selected for season of the dry.

After conducting visual assessments, we utilized camera traps and digital cameras to capture images of hippo pods, which we subsequently counted in the field to ensure alignment with the visual tallies made in the area. Throughout these visual assessments, we dedicated more time to record during the wet season than in the dry season, as hippos were frequently submerged and more difficult to count when river levels rose in the wet months. To minimize the likelihood of counting inaccuracies, the pods of hippo and counting sites were reviewed multiple times.

During the total count, the sex and age composition of every hippopotamus sighting were recorded. The sexes of the animals were determined by size of the body and sexual dimorphic features, while age classes were determined based on body size, their positions, and external features. This follows the approach employed by Ref. [19]. Individuals were classified as adult, sub adult, or young based on age. Individuals are regarded as belonging to the same category when distances between them are less than 50 m [20], they react to environmental stimuli similarly, and they travel in the same direction as other groups. The total number of hippos recorded was combined to determine the hippo population size in the DWS.

The main drawbacks of this approach are its lengthy duration. The observer's counting ability may be affected by encountering hazardous animals like lions and crocodiles while walking along the riverbanks in the DWS, leading to potential frustration and bias in the data record. Even with the wet season causing high water levels and low visibility, the data record could have been affected. Hence, the researchers repeated and compared the counts to reach a consensus among the observers.

### 2.1 Distribution and habitat suitability of hippo

The DWS is characterized by heterogeneous habitats. In each count, four habitat types (woodland, riverine forests, seasonal flooded grassland, and savanna grassland) were identified based on landscape vegetation structure and terrain, with hippos observed in these areas (Table S2) [21,22]. The habitats were categorized by merging pictures from 30 m-resolution LANDSAT with a Google Earth and Shape file generated using GPS data outlining the boundaries of each habitat from ground truth information. Since it would be impossible to cover every riverbank, four habitat types were selected for assessment of suitability based on their size, proximity to human settlement, and livestock disturbance [23]. States that, the habitat suitability of the surrounding buffer zone, which is dependent on three main criteria, determines the distribution and habitat use of hippos. These are the proximity of grazing grounds, their elevation and slope, human settlement, and cattle disturbance. Multiple-ring buffer analysis was used using geographic information system (Arc GIS) 10.2 to classify and reclassify grazing site suitability based on livestock disturbance, settlement, and proximity to resting water. Based on research in the field and literature, the slope class that facilitates hippo migration to the land was determined [24] (Table 2).

**Table 2:** Suitability classes for each criteria/factor of hippopotamus habitat.

Criteria	Suitability class			Source
	Highly suitable	Moderately suitable	Not suitable	
Elevation	1634–1900m	1900–2000m	>2000m	[24]
Grazing land proximity	2–5 km	5–10 km	>10	[23]
Slope	2°	2–7°	>7°	[24]
Settlement disturbance	5.5 km	3 km	0.5 km	[25]
Water depth	1.5–2m	2–3m	>3m	[25]

The Dhidhessa River was encompassed by one of these ecosystems, influencing the availability of forage (Table S2). The habitat preference of the hippos was assessed considering their density across four kinds of ecosystem in both seasons [26,27]. Research conducted by Refs. [26,27] indicated that hippos exhibited preferences for certain habitats, with some areas hosting significantly higher populations than others. Non-direct evidence such as droppings, signs of feeding, footprints, and pathways were utilized as indicators for evaluating habitat preferences in the research area [28]. Understanding the factors that affect the Preferences for habitat of hippos throughout different seasons was crucial, which necessitated conducting research in both dry and wet periods to account for major differences in vegetation cover [29].

### 2.2 Data analysis

Origin Pro8.5.1, IBM SPSS version 26, and Microsoft Excel were used to examine the size of the population and dispersion density of hippopotamuses. The significance of population size differences between sites, seasons, and habitats was analyzed using chi-square and t-tests. To evaluate the adequacy of the habitat, multiple-ring buffer analysis was used in Arc GIS 10.2. To make the combination compatible, the data class is converted into a consistent measurement of its appropriateness. Based on literature and field observations, each layer was classified into three categories of suitability: high suitability, moderate suitability, and not suitability. In addition, the degree of relevance of 0.05 was used as the standard for comparing the average and confidence intervals of 95 %.

## 3 Results

### 3.1 Population size and structure

On average, 182 ± 7.86 individual hippos were counted in DWS. The average density of hippo in the study area were 0.09 ± 0.004 individuals/ha. In the field of our study, the estimated numbers of hippopotamuses in both the rainy and dry seasons were 231 ± 8.93 and 133 ± 6.8 individuals, respectively (Fig. S1). This phenomenon may be linked to the decrease in river volume in the season of dry, causing hippos to cluster in wider gorges and slower-flowing parts of the river. The number of hippos recorded during the two seasons was significantly different (χ2 = 26.4, df = 1, P < 0.05). Hippos were found in greater numbers at the Sefera Tabia site (24 %, n = 88), while the Bedhassa site recorded the fewest hippos (11.8 %, n = 43).Nevertheless, there was no notable variation in the hippo counts across various locations during both the wet and dry seasons (χ2 = 5.44, df = 5, p > 0.05) (Table 3).

**Table 3:** Population size of hippopotamus in DWS during 2021/2022 (Mean ± SE).

Counting site	Area/ha	Buffer zone	Dry season	Wet season	Mean	Density/ha
Turee	1003.47	Planation farm and riverine	35 ± 0.63	28 ± 1.06	31.5 ± 0.84	0.03 ± 0.001
Kerkeha	286.67	Planation farm, riverine and natural forest	42 ± 0.63	20 ± 0.39	31 ± 0.51	0.11 ± 0.002
Abote	70	Natural forest and planation farm	45 ± 1.18	18 ± 0.76	31.5 ± 0.97	0.45 ± 0.014
Bedasa	253.35	Planation farm and riverine	30 ± 1.55	13 ± 1.67	21.5 ± 1.61	0.08 ± 0.01
Loko	13.65	Grassland	26 ± 2.29	19 ± 0.57	22.5 ± 1.43	1.65 ± 0.10
Sefera Tabia	350.75	Planation farm and riverine	53 ± 2.65	35 ± 2.35	44 ± 2.5	0.13 ± 0.01
Total/average	1977.9		231 ± 8.93	133 ± 6.8	182 ± 7.86	0.09 ± 0.004

The age structure of the population of hippo in DWS ecosystem is presented in Table 4. Of the individuals observed, 62.08 % were adults, 20.9 % sub-adults and 17.03 % were calves (Fig.S1, Fig.S2). Our analysis indicated a notable difference in the age categories of hippos across the two seasons (t = 3.3, df = 4, p < 0.05). 83 % of the hippopotamus populations were adults, while 17 % were young hippos. Among adults, 33.8 % were males and 49.2 % were females. Females are by far larger than males (t = 4.9, df = 1, P = 0.06). The number of adult males were nearly significantly higher than calves (t = 4.6, df = 1, p = 0.06) and there was no significant variation between in the number of adult females and calves recorded in the two season (t = 2.5, df = 1, p = 0.12). The sex ratio of adult male to adult female was 1.00:1.45. On other hand, adult to calves ratio were 4.95:1.00 (Table 4).

**Table: 4:** Age distribution and sex classes of hippo population in DWS in two seasons (Mean + SE) (AM = Adult male, AF = Adult female, SAM= Sub adult male, SAF= Sub adult female, C= Calves and A = Adult).

Age structure and sex ratio			
	Season		
Categories	Dry	Wet	Mean
AM	60 ± 3.08	33 ± 1.43	46.5 ± 2.26
AF	85 ± 8.66	48 ± 4.78	66.5 ± 6.7
SAM	17 ± 6.55	13 ± 3.04	15 ± 4.79
SAF	28 ± 4.06	18 ± 1.92	23 ± 2.99
C	41 ± 1.16	21 ± 3.21	31 ± 2.19
Ratio			
AM:AF	00:01.4	1.00:1.5	1.00:1.45
C:A	00:04.6	1.00:5.3	1.00:4.95
AF:C	2.1:1.00	2.3:1.00	2.2:1.00
SA:C	1.00:1.00	1.47:1.00	1.2:1.00

**Fig. 2:**
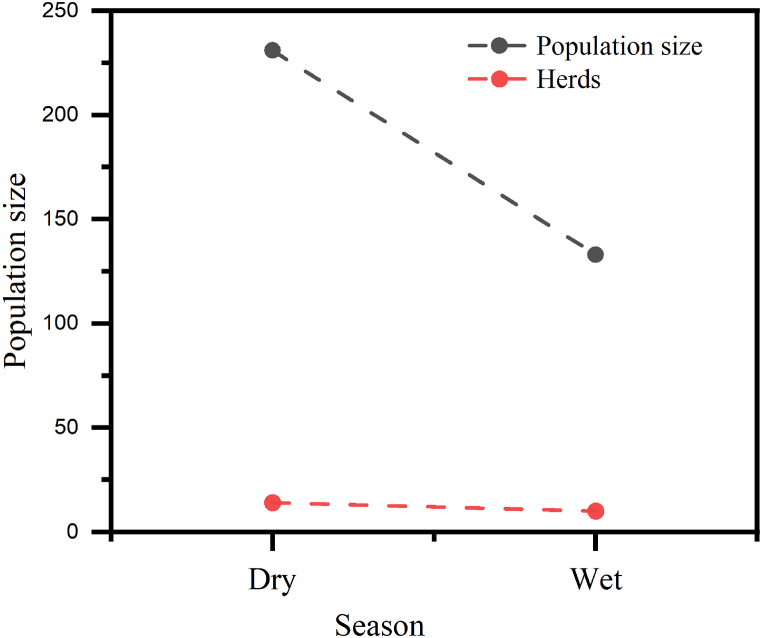
Population size and herds of hippo in study area.

Population size groups were assessed for 133 individuals in 10 herds during the wet season and 231 individuals in 14 herds in the dry season. Both the number of individuals and herds were decreased during wet season (Fig. 2).

#### 3.1.1 Slope suitability

**Fig. 3:**
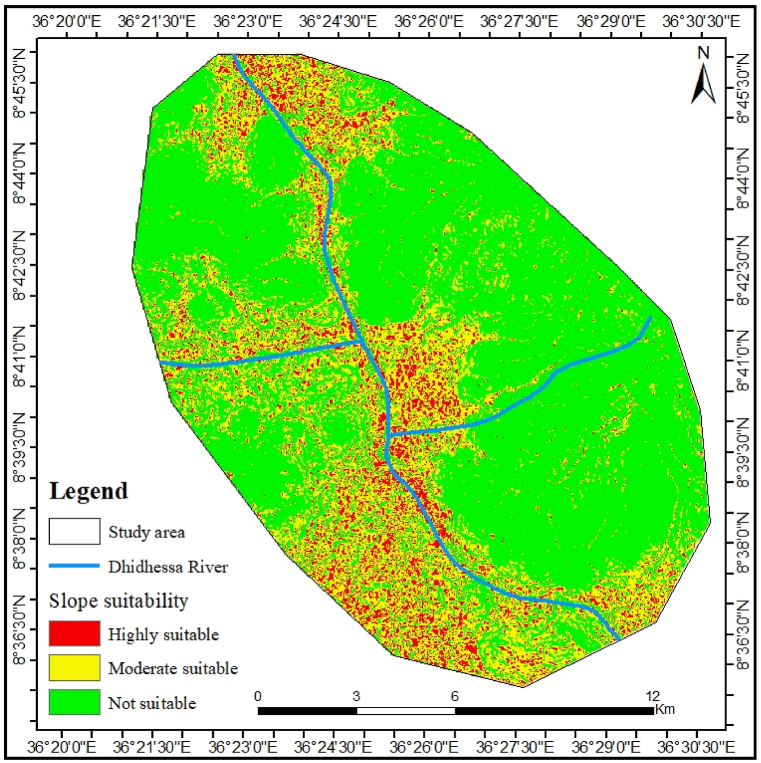
Slope suitability classes of hippopotamus habitat.

The habitats within the DWS and nearby areas were classified according to their suitability for hippos, ranging from highly suitable to poorly suitable (see Table S3). The slope classification, which is crucial for hippos to access land, was established through a combination of literature review and on-site observations. Due to their size and body structure, hippos struggle to ascend steep hills. Consequently, the slope values deemed appropriate are those that facilitate movement for this species. Only 7.95 % of the assessed slope classes are classified as highly suitable, 26.32 % are considered moderately suitable, and 65.72 % are regarded as unsuitable (i.e., the slope classes that hippos cannot traverse)(Fig. 3).

#### 3.1.2 Grazing ground proximity

**Fig. 4:**
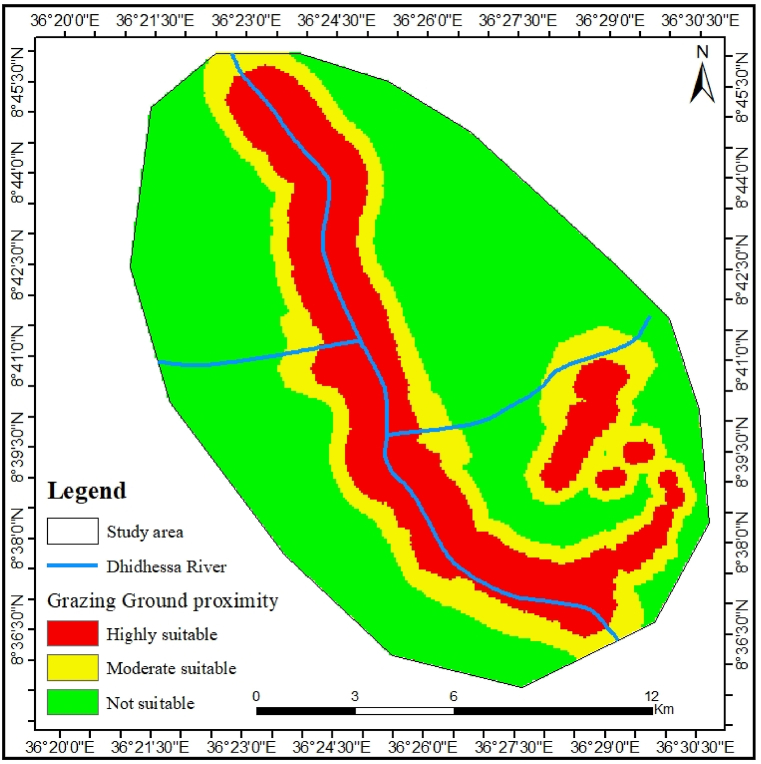
Hippopotamus grazing ground proximity classes to the Dhidhessa River.

In DWS, hippos were found primarily near water bodies that were open, slow-moving, and had enough depth for them to submerge their large bodies while accessing forage along the riverbanks. As shown through multiple rings buffer analysis, the area’s most ideal for hippos' nighttime grazing tend to be in proximity to water for rest. Thus, while maintaining other variables constant and considering the hippos' ability to travel and graze, only 23.18 % of the approximate area was determined as highly suitable, 18.49 % as moderately suitable, and the remaining 58.31 % as unsuitable (Fig. 4).

#### 3.1.3 Settlements disturbance

**Fig. 5:**
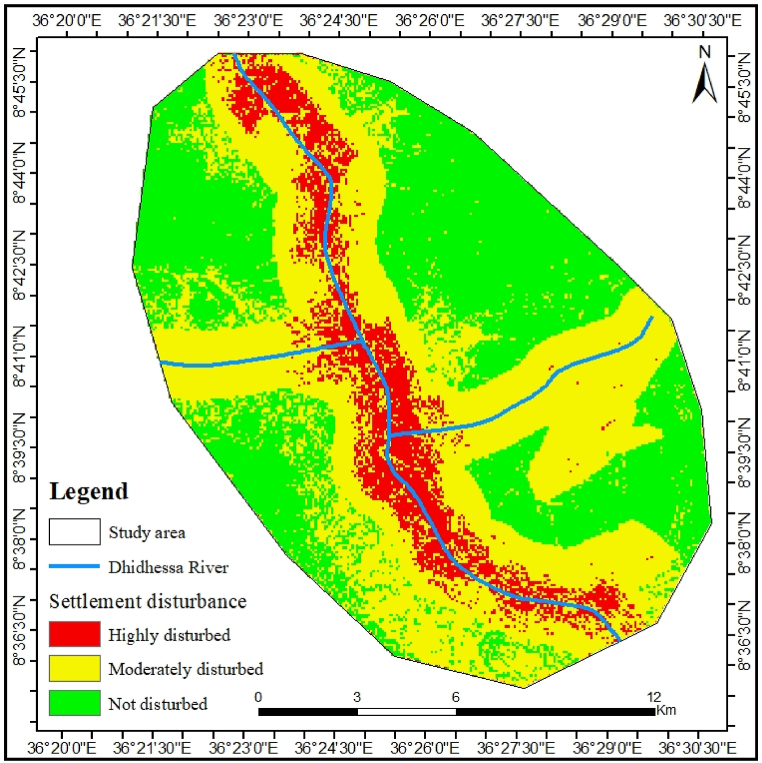
Settlements disturbance on hippopotamus habitat.

In addition to the cultivation of grazing land for hippos and competition with livestock in the same region, permanent settlements significantly disrupt their habitat (Fig. S3). Observations from field visits indicate that residents living near the river constructed various barriers to keep these animals from entering their gardens. The manmade obstacles threatening the survival of these animals included holes dug for this purpose and stone fences. Residents were aware that, due to the hippo's large size and short legs, it could not navigate these barriers. From the settlements, multiple buffering rings were created at designated distances surrounding the input feature, as shown in Fig. 5, which illustrated the considerable human and livestock disturbance affecting hippo habitats in the DWS. In the assessed terrestrial habitats of the chosen locations, only 19.8 % of the land was free from human interference, while 46.3 % of the land experienced significant disturbances detrimental to hippos' survival in the region. Conversely, 33.9 % of the area was classified as having moderate disturbances.

#### 3.1.4 Water depth suitability

**Fig. 6:**
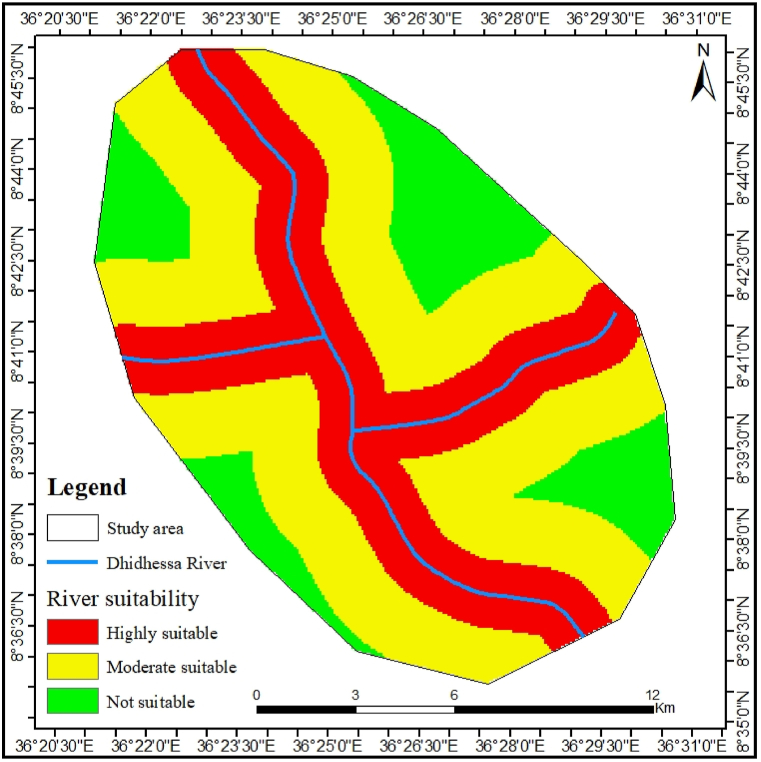
Water suitability classes of Dhidhessa River.

Hippos are affected by their choice of habitat, the characteristics of the shoreline, as well as the depth and current of the water. Most hippo groups are located in areas with relatively stable, gently sloping banks and smooth flowing waters where they can stand and kneel on the bottom while staying close to the surface for breathing, allowing young to nurse easily. Consequently, 32.56 % of river regions are considered highly suitable, whereas 48.78 % and 18.66 % are categorized as moderately suitable and unsuitable, respectively (Fig. 6).

**Fig. 7:**
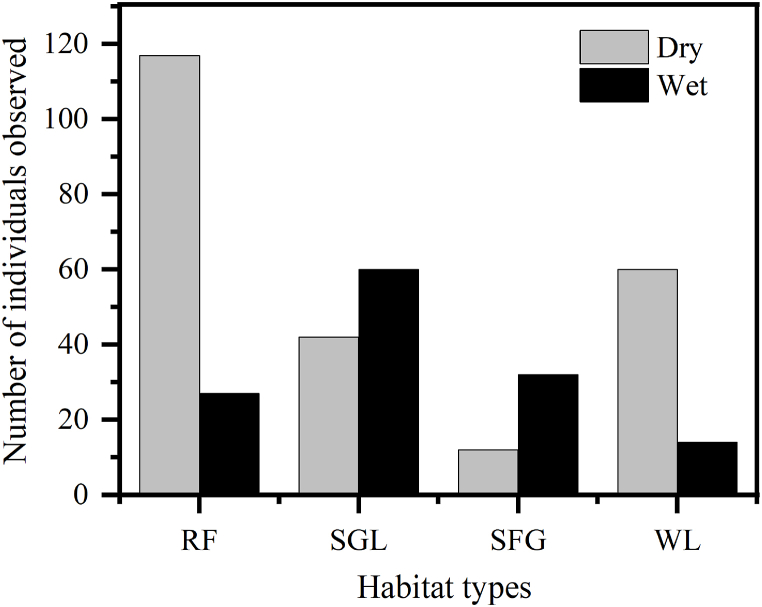
Habitat preference of hippopotamus in study area. Key:RF= Riverine forest, SGL= Savanna grassland, SFG = seasonally flooded grassland, WL= Woodland.

The wetland functions as both a feeding ground and a buffer zone, accommodating various types of habitats. During dry weather, hippos displayed a strong preference for riverine vegetation. Of the total, 50.6 % (n = 117) of the hippos utilized riverine habitats during the dry season, whereas only 20.3 % (n = 27) did so in the wet season. In contrast, during the wet season, 45.1 % (n = 60) of hippos occupied savannah grassland, compared to 18.2 % (n = 42) in the dry season (Fig. S4). In the two seasons of the study period, the use of each type of habitat was significantly different (χ2 = 76.3, df = 3, P < 0.05) (Fig. 7).

## 4 Discussion

### 4.1 Population size and structure

The DWS comprises several kinds of ecosystems that provide suitable living habitats for common hippos. Compared to the previously reported hippo populations in other locations such as Kainji Lake National Park [30] and Lake Kawara [31] in Nigeria [32], and Boye wetland [8] and Upper Dhidhessa River in Ethiopia [12], the number of hippo recorded in these study regions was relatively high. This could be because the Dhidessa River is the third- biggest river in the country. The lower part of the river (the flat plains and extended grasslands) is the best habitat for hippos, and the Dhidhessa River depth provides shelter for hippos from high temperatures in the Dhidhessa valley, particularly during the dry season. The mean population of hippos at Faro National Park, along with adjacent hunting zones in North Cameron (685 individuals) [33], and Chebera Churchura National in Ethiopia (456 individuals) [11], is notably higher than the current figures. This disparity may be ascribed to the fact that Faro National Park and Chebera Churchura National Park are designated protected areas where conservation measures have been actively enforced to safeguard wildlife and their habitats, in contrast to the human- dominated DWS. Furthermore, records of hippos were obtained from nine lakes and rivers within Chebera Churchura National Park, which may explain the increased number of hippos reported. Historically, the DWS has hosted the largest hippos' population in the country. The main reason for the region's former high population status may be the fact that these regions serve as national parks. In addition, the resettlement activities, and the recently introduced Dhidhessa sugar factory have occupied a large part of the suitable habitat for hippos and other wildlife. Based on this, an inappropriate agricultural activity has fragmented the continuous habitat and range routes of hippos, while increasing the conflict of human hippo in DWS and its surroundings [34]. Similar circumstances have been reported in other parts of Ethiopia [15], in Kenya [35,36], and Democratic Republic of Congo [37].

In the DWS, the dry season saw a higher number of hippos than the rainy season. This phenomenon may be due to the decrease in river volume in the dry season, leading hippos to cluster in broader gorges and slower-moving sections of the river. Consequently, this has enhanced the precision of hippo counts in the dry season [12,14]. Nevertheless, this variation in seasonal abundance is contrary to the anticipated pattern, where higher numbers would be expected in the wet season, as resource availability typically increases following rainfall [11,38,39].The likely explanation for the greater number of individuals observed during the dry season may be linked to three key factors: the rising levels of river water, changes in the intensity of anthropogenic activities such as farming, and the increased complexity of vegetation structure. In the wet season, human and livestock intrusion into the DWS is more significant since the surrounding regions are filled with crops, leading to reduced visibility due to the falling savannah grass, which makes counting hippos challenging [12,14,and22]].

Each animal population should have a balanced age structure to maximize productivity and population viability [40]. Likewise, unequal gender and age structures can affect the population growth rate [41] and the future fate of the population. The significant presence of female in the current research area is quite encouraging, and the population is healthy and growing. This may be due to external causes such as predators that hamper animal reproductive capacity during the season, and observer count skills. Similarly, the ratio of female adults to males was relatively high. This is also encouraging. On the other hand, the ratio of juveniles to adults was unpredictable during both seasons. Similar findings have been cited elsewhere [8,11,42,and^43^]].

### 4.2 Habitat suitability and distribution

Hippos have significantly higher concentrations in some environments than in others, indicating habitat preferences [44]. According to Ref. [45], the slopes of the area significantly affect the movement of hippo and access to suitable habitats. The outcomes of the present DWS have lower slope suitability rate (7.95 %) than the previous studies (36.77 %) in Chebera Churchura National Park, Ethiopia [11]. Due to its size and structure, the species could not ascend steep hills, leading to recommended slope values that were suitable for its travel conditions. Consequently, the digital altitude shows that the altitude is very low from 1280 to 1350 m above sea level (Table S2), and that the river's coast gradient below 7° and is suitable for the animal in the DWS.

Hippo prefers to graze at night, and the best place to do so is near the water where they can rest [14]. As a result, some of the areas of the DWS are suitable, and 58.31 % of the areas were unsuitable. [9], and [11], reported similar observation, indicating that DWS still provides the suitable habitat for hippo conservation with minimal efforts. Furthermore, the areas considered inappropriate during the study were shared by domestic animals. Field trips have proven that residents living near the river have built a variety of obstacles to prevent animals from entering the gardens. The stone walls and holes created by this reason are human barriers that jeopardize the life of this animal. They were aware that because of its large size and small leg, it could not get through such obstructions. Such access inhibition techniques have been mentioned somewhere [9]. Unlike others, hippo is not completely free of human interference as the enforcement in the DWS is minimal. But, this outcome is relatively low compared to the conclusion of the [11], which indicates that 31.72 % of the land area in Chebera Churchura National Park, Ethiopia, was unaffected by human activities.

Multiple buffers of rings at a specific distance from settlements that enclose the input features in the research area showed that they were very disturbed and inappropriate for hippo. The results of the current research area clearly show that there has been significant severe disturbance of permanent settlements, not only on the grazing ground, but also leading to competition with domestic animals, thus significantly disturbing the hippo's habitat. The present results are comparatively consistent with those of [9], who found that 50.88 % of the studied regions experienced high levels of disturbance, rendering them unsuitable for hippos in Lake Tana, Ethiopia. However, this result is relatively lower than the findings of [11], which indicate that in the Chebera Churchura National Park, Ethiopian, 31.72 % of the land area is free from human intervention.

In DWS, the Dhidhessa River has a higher water depth suitability rate for hippo than some previous studies in Lake Tana, Ethiopia [9]. This may be explained by the Dhidhessa River's depth and size, which provided hippos with excellent protection from the hot weather in the Dhidhessa valley, particularly during the dry season. In addition, the DWS's neighboring banks featured vast grassland plains that would give hippos' adequate range. Similar results were reported by Ref. [14].

The selection of animal habitat reflects a strategy that improves survival and successful reproduction [46]. Hippos use different habitats in different seasons to maximize the gain, and in the present study area, resources are not evenly distributed across all habitats during any season. For this reason, seasonal resource fluctuations have been reported to regulate animal movement between habitats repeatedly [47,48]. Similarly, the amount of green grass in the area, the amount of rainfall, water levels and plant phenology have all affected how the seasonal habitat of hippo changes in the study area. For example, hippos frequently utilized the riverine and wooded habitats in the DWS in the dry season. Because of this, grass dries out during the dry season. As the grasses in the savanna grassland habitat grew towards the end of the rainy season, this habitat was used sparingly. In a similar way, during the start of the wet season, this habitat acts as a refuge for hippo herds moving to highland habitats. In addition, the grassland with isolated trees that had burned during the dry season had lush green swards in the wet season. Comparable results have been obtained in the Yes, the listing is correct Shire River in Malawi [49], Queen Elizabeth National Park in Uganda [50], and Chebera Churchura National Park in Ethiopia [11].

## 5 Conclusions and away forward

The goal of this research was to ascertain the hippo population and the suitability of their habitat in the DWS. A total of 231 and 133 hippos were recorded in DWS in both dry and rainy seasons, respectively. Of these, 62.08 % were adults, 20.88 % were sub-adults and 17 % young. The study showed that DWS remains the most important fortress and reliable for the conservation of the species. Furthermore, the study shows that if a complete survey is conducted throughout the river route and at night, the overall figure could be much better. Hippo habitat associations and distribution vary greatly between habitats, seasonal and other environmental conditions, which is a very important area for conservation. Assessing each element based on habitat suitability categories leads to differing outcomes. In the DWS, the high degrees of disruption from nearby grazing areas and human activities adversely affect hippo habitats, making many regions unsuitable. For example, it was found that 58.31 % of the regions were inappropriate due to the closeness of grazing grounds, while disturbances from settlements accounted for 46.27 % of areas being considered unsuitable.

Hippo habitats need to be protected and linked to adjacent safeguarded zones like the Arjo-Diga protected forest [51] and the Abba Diko controlled hunting area through landscape connectivity to guarantee the long-term hippos' ability to survive in the DWS. In the potential hippo habitats within the DWS, agricultural expansion, human settlements, investments, and competition with livestock will not be considered [52]. Future management and conservation initiatives should also focus on finding solutions to human-caused issues like habitat fragmentation by creating suitable corridors within the DWS to link the separated patches of suitable hippos' habitat. Finally, the Ethiopian Wildlife Authority and regional government therefore recommend revitalizing the status of the DWS, which should be declared a national park with new management plans.

## CRediT authorship contribution statement

**Girma Gizachew Tefera:** Writing – review & editing, Writing – original draft, Methodology, Investigation, Funding acquisition, Formal analysis, Data curation, Conceptualization. **Tadesse Habtamu Tessema:** Writing – review & editing, Visualization, Supervision, Resources, Methodology, Conceptualization. **Tibebu Alemu Bekere:** Writing – review & editing, Visualization, Supervision, Conceptualization. **Tariku Mekonnen Gutema:** Writing – review & editing, Supervision, Methodology, Investigation, Formal analysis, Conceptualization.

## Ethical approval

The research proposal was reviewed and granted approval by the Review Board at 10.13039/501100005068Jimma University (JU) College of Agriculture, Veterinary, and Medicine. The vice president's office also reauthorized and accepted the request for research and community service from the review board. JU sanctioned the process for collecting consent needed for the data collection tools (RGS/752/2021). Following approval from the human ethics research committee, we received an authorization letter along with a request for collaboration from all Kebeles and villages to carry out this research project near Dhidhessa Wildlife Sanctuary.

## Availability of data

Data included in article will be obtained from the first author upon request.

## Funding

The author(s) received no specific funding for this work.

## Declaration of competing interest

The authors declare that they have no known competing financial interests or personal relationships that could have appeared to influence the work reported in this paper.
