# Supplementary material for: Population status and habitat suitability of the vulnerable common hippopotamus (Hippopotamus amphibius) in the Dhidhessa Wildlife Sanctuary, Southwestern Ethiopia
Source: Heliyon. 2024 Nov 8;10(22):e40186. doi: 10.1016/j.heliyon.2024.e40186 (PMC11600075; doi:10.1016/j.heliyon.2024.e40186)
Supplement: Multimedia component 1 [file mmc1.docx]

**Supplementary**
Name of the data collector----------------------------------------- Date--------------- Survey site/transect---------Direction of river_________ area of river_____ (ha) Altitude-----------Temperature (C^o^)-------- Season--------------Weather condition------------Starting time---------------Stopping time------- Signature----------------
Key: (Sd=Sight distance, Sa= Sight angle Am=Adult male Sam= Sub adult male You= Young
Af= Adult female Saf= Sub adult female Un = Unknown)

**Table S1: Data sheet of hippopotamus population census**

| No of observation | Observation time | Sight dis & angle | | Sex & age category | | | | | | Total | Herd  size | Habitat  type | Activity |
| --- | --- | --- | --- | --- | --- | --- | --- | --- | --- | --- | --- | --- | --- |
|  |  | Sd | Sa | Am | Sam | You | Af | Saf | Un |  |  |  |  |
|  |  |  |  |  |  |  |  |  |  |  |  |  |  |
|  |  |  |  |  |  |  |  |  |  |  |  |  |  |
|  |  |  |  |  |  |  |  |  |  |  |  |  |  |
|  |  |  |  |  |  |  |  |  |  |  |  |  |  |
|  |  |  |  |  |  |  |  |  |  |  |  |  |  |
|  |  |  |  |  |  |  |  |  |  |  |  |  |  |
|  |  |  |  |  |  |  |  |  |  |  |  |  |  |
|  |  |  |  |  |  |  |  |  |  |  |  |  |  |
|  |  |  |  |  |  |  |  |  |  |  |  |  |  |
|  |  |  |  |  |  |  |  |  |  |  |  |  |  |
|  |  |  |  |  |  |  |  |  |  |  |  |  |  |

**Table S 2: Habitat suitability data in in the study area**

| No | **Easting** | **Northing** | **Names** | **Elevation** |
| --- | --- | --- | --- | --- |
| 1 | 224888 | 957041 | Woodland | 1533 |
| 2 | 225080 | 956495 | Woodland | 1483 |
| 3 | 225080.66 | 956449 | Woodland | 1483 |
| 4 | 224787.9 | 955958.6 | Woodland | 1486 |
| 5 | 224661 | 955696.9 | Woodland | 1475 |
| 6 | 224522 | 955379.5 | Woodland | 1452 |
| 7 | 224090.7 | 955001 | Woodland | 1438 |
| 8 | 223682 | 954709 | Woodland | 1442 |
| 9 | 222973.7 | 954511 | Woodland | 1370 |
| 10 | 222939.868 | 954383 | Woodland | 1364 |
| 11 | 222670 | 954240.79 | Woodland | 1351 |
| 12 | 222082 | 953610 | Woodland | 1312 |
| 13 | 221414.5 | 953727.5 | Woodland | 1297 |
| 14 | 221069.926 | 953860 | Woodland | 1304 |
| 15 | 220578 | 954027.8 | Woodland | 1316 |
| 16 | 220207 | 954236.59 | Woodland | 1327 |
| 17 | 219753.97 | 954395.49 | Woodland | 1321 |
| 18 | 219347.97 | 954789.757 | Woodland | 1320 |
| 19 | 218290.59 | 955932 | Woodland | 1290 |
| 20 | 218157 | 956117 | Woodland | 1287 |
| 21 | 218019 | 956409 | Woodland | 1305 |
| 22 | 222076 | 958091 | Woodland | 2471 |
| 23 | 224215 | 957848 | Woodland | 2454 |
| 24 | 222965 | 959740 | Woodland | 2471 |
| 25 | 222884 | 959499 | Woodland | 2472 |
| 26 | 222935 | 959090 | Woodland | 2472 |
| 27 | 222298 | 958932 | Woodland | 2472 |
| 28 | 222729 | 958925 | Woodland | 2472 |
| 29 | 222539 | 958668 | Woodland | 2473 |
| 30 | 222246 | 958479 | Woodland | 2473 |
| 31 | 222076 | 958091 | Woodland | 2451 |
| 32 | 223881 | 957789 | Woodland | 2443 |
| 33 | 221922 | 957991 | Woodland | 2475 |
| 34 | 224215 | 957848 | Woodland | 2443 |
| 35 | 221911 | 957704 | Woodland | 2458 |
| 36 | 221840 | 957624 | Woodland | 2473 |
| 37 | 221720 | 957335 | Woodland | 2451 |
| 38 | 223384 | 957061 | Woodland | 2451 |
| 39 | 222991 | 957010 | Woodland st | 2453 |
| 40 | 221537 | 957186 | Woodland | 2447 |
| 41 | 212685.7239 | 968577.798 | Riverine forest | 1257 |
| 42 | 212491.0281 | 968531.9775 | Riverine forest | 1252 |
| 43 | 212580.7193 | 968246.0185 | Riverine forest | 1285 |
| 44 | 212669.9927 | 968068.7709 | Riverine forest | 1295 |
| 45 | 212679.1665 | 968017.2667 | Riverine forest | 1300 |
| 46 | 212800.2693 | 967918.4287 | Riverine forest | 1275 |
| 47 | 212845.3497 | 967881.6364 | Riverine forest | 1241 |
| 48 | 212909.9801 | 967859.8989 | Riverine forest | 1275 |
| 49 | 212989.7779 | 967839.7925 | Riverine forest | 1309 |
| 50 | 213074.7481 | 967841.2781 | Riverine forest | 1272 |
| 51 | 213160.5639 | 967845.3279 | Riverine forest | 1349 |
| 52 | 213336.3287 | 967856.7249 | Riverine forest | 1386 |
| 53 | 213392.8357 | 967840.0999 | Riverine forest | 1300 |
| 54 | 213431.1015 | 967827.2033 | Riverine forest | 1100 |
| 55 | 213483.0943 | 967793.5389 | Riverine forest | 1200 |
| 56 | 213569.5335 | 967685.7743 | Riverine forest | 1300 |
| 57 | 213626.1715 | 967550.1473 | Riverine forest | 1250 |
| 58 | 213636.4941 | 967354.1179 | Riverine forest | 1400 |
| 59 | 213665.5453 | 967035.4269 | Riverine forest | 1280 |
| 60 | 213733.9067 | 966928.1979 | Riverine forest | 1050 |
| 61 | 213974.05 | 966884.7484 | Riverine forest | 1052 |
| 61 | 214174.5307 | 966781.4019 | Riverine forest | 1056 |
| 62 | 214486.1244 | 966515.7003 | Riverine forest | 1048 |
| 63 | 214708.4361 | 966328.0661 | Riverine forest | 1090 |
| 64 | 214782.0281 | 966224.7093 | Riverine forest | 1136 |
| 65 | 214756.6689 | 965990.5047 | Riverine forest | 1320 |
| 66 | 214730.6777 | 965797.8753 | Riverine forest | 1275 |
| 67 | 214661.8006 | 965685.8815 | Riverine forest | 1284 |
| 68 | 214456.0821 | 965512.5779 | Riverine forest | 1256 |
| 69 | 214262.6623 | 965210.5245 | Riverine forest | 1248 |
| 70 | 214252.0329 | 965064.2399 | Riverine forest | 1245 |
| 71 | 214294.8193 | 964886.5203 | Riverine forest | 1300 |
| 72 | 214371.0213 | 964751.9159 | Riverine forest | 1302 |
| 73 | 214370.3227 | 964543.6837 | Riverine forest | 1306 |
| 74 | 214326.1065 | 964222.3677 | Riverine forest | 1305 |
| 75 | 214326.1065 | 964061.2239 | Riverine forest | 1324 |
| 76 | 214351.6883 | 963918.2129 | Riverine forest | 1325 |
| 77 | 214453.022 | 963683.3612 | Riverine forest | 1320 |
| 78 | 214406.5431 | 963278.4847 | Riverine forest | 1130 |
| 79 | 214457.7359 | 963115.7053 | Riverine forest | 1209 |
| 80 | 214510.3471 | 962942.9231 | Riverine forest | 1082 |
| 81 | 214538.7005 | 962891.9631 | Riverine forest | 1120 |
| 82 | 214774.1981 | 962662.8265 | Riverine forest | 1203 |
| 83 | 214859.6107 | 962449.2235 | Riverine forest | 1230 |
| 84 | 214974.459 | 962347.907 | Riverine forest | 1304 |
| 85 | 215290 | 961924 | Riverine forest | 1369 |
| 86 | 215316.217 | 961513.9625 | Riverine forest | 1302 |
| 87 | 215536.3695 | 961036.4437 | Riverine forest | 1245 |
| 88 | 215566.9097 | 960692.282 | Riverine forest | 1256 |
| 89 | 215677.4755 | 960132.4104 | Riverine forest | 1246 |
| 90 | 215734.7827 | 960005.5265 | Riverine forest | 1247 |
| 91 | 215828.2474 | 959860.3731 | Riverine forest | 1245 |
| 92 | 215841 | 959623 | Riverine forest | 1246 |
| 93 | 216027.1926 | 958905.6358 | Riverine forest | 1230 |
| 94 | 216184 | 958541 | Riverine forest | 1232 |
| 95 | 216195.0471 | 958408 | Riverine forest | 1230 |
| 96 | 216122 | 958112 | Riverine forest | 1236 |
| 97 | 215911.1733 | 957928.1509 | Riverine forest | 1248 |
| 98 | 215857 | 957804 | Riverine forest | 1295 |
| 99 | 215883 | 957664 | Riverine forest | 1296 |
| 100 | 215923 | 957569 | Riverine forest | 1296 |
| 101 | 216034 | 957460 | Riverine forest | 1400 |
| 102 | 216155 | 957413 | Riverine forest | 1500 |
| 103 | 216200 | 957368.9574 | Riverine forest | 1600 |
| 104 | 216207 | 957351 | Riverine forest | 1671 |
| 105 | 216234 | 957325 | Riverine forest | 1820 |
| 106 | 216262 | 957317 | Riverine forest | 1563 |
| 107 | 216284.3083 | 957323.6925 | Riverine forest | 1563 |
| 108 | 216320.7306 | 957347.3767 | Riverine forest | 1423 |
| 109 | 216343.3861 | 957347.3767 | Riverine forest | 1453 |
| 110 | 216369.2448 | 957347.3767 | Riverine forest | 1630 |
| 111 | 216427 | 957329 | Riverine forest | 1425 |
| 112 | 216510 | 957282 | Riverine forest | 1236 |
| 113 | 216760.877 | 957144.846 | Riverine forest | 1302 |
| 114 | 217093 | 956915 | Riverine forest | 1203 |
| 115 | 217305 | 956674 | Riverine forest | 1230 |
| 116 | 217476 | 956333 | Riverine forest | 1400 |
| 117 | 217773.211 | 956139.3119 | Riverine forest | 1402 |
| 118 | 218036.409 | 955968.604 | Riverine forest | 1403 |
| 119 | 218070.061 | 955800.431 | Riverine forest | 1405 |
| 120 | 218053 | 955421 | Riverine forest | 1423 |
| 121 | 218101 | 955235 | Riverine forest | 1426 |
| 122 | 218065.4044 | 955027.0801 | Riverine forest | 1560 |
| 123 | 218262.5364 | 954993.3314 | Riverine forest | 1563 |
| 124 | 218305.3625 | 955243.4859 | Riverine forest | 1523 |
| 125 | 218254.1444 | 955441.9561 | Riverine forest | 1554 |
| 126 | 218254.1444 | 955660.9715 | Riverine forest | 1563 |
| 127 | 218254.1444 | 955960.0419 | Riverine forest | 1506 |
| 128 | 218198.0199 | 956102.1684 | Riverine forest | 1800 |
| 129 | 217882.2249 | 956306.9902 | Riverine forest | 1820 |
| 130 | 217630.5265 | 956471.0184 | Riverine forest | 1863 |
| 131 | 217472.2123 | 956786.7209 | Riverine forest | 1100 |
| 132 | 217226.9739 | 957065.5061 | Riverine forest | 1102 |
| 134 | 216813.183 | 957351.8705 | Riverine forest | 106 |
| 135 | 216507.4555 | 957513.2804 | Riverine forest | 1023 |
| 136 | 216377.4282 | 957554.6528 | Riverine forest | 1023 |
| 137 | 216306.9295 | 957544.153 | Riverine forest | 1506 |
| 138 | 216265.6093 | 957584.594 | Riverine forest | 1504 |
| 139 | 216144.7708 | 957631.5313 | Riverine forest | 1503 |
| 140 | 216091.7574 | 957683.5895 | Riverine forest | 1500 |
| 141 | 216070.2251 | 957734.7285 | Riverine forest | 1520 |
| 142 | 216064.8356 | 957780.2223 | Riverine forest | 1563 |
| 143 | 216076.503 | 957806.9608 | Riverine forest | 1452 |
| 144 | 216301.0441 | 958002.7693 | Riverine forest | 1456 |
| 145 | 216397.0733 | 958391.8973 | Riverine forest | 1453 |
| 146 | 216380.799 | 958587.8304 | Riverine forest | 1450 |
| 147 | 216200.8057 | 959032.8313 | Riverine forest | 1100 |
| 148 | 216039.634 | 959653.7951 | Riverine forest | 1120 |
| 149 | 216025.1144 | 959924.0596 | Riverine forest | 1130 |
| 150 | 215910.9573 | 960101.3487 | Riverine forest | 1120 |
| 151 | 215869.2249 | 960193.7485 | Riverine forest | 1152 |
| 152 | 215765.1849 | 960720.5754 | Riverine forest | 1144 |
| 153 | 215732.5203 | 961088.6763 | Riverine forest | 1136 |
| 154 | 215513.4335 | 961563.8838 | Riverine forest | 1125 |
| 155 | 215485.8118 | 961995.8916 | Riverine forest | 1750 |
| 156 | 215122.4253 | 962484.0755 | Riverine forest | 1796 |
| 157 | 215027.6329 | 962567.6992 | Riverine forest | 1752 |
| 158 | 214959.902 | 962737.0832 | Riverine forest | 1620 |
| 159 | 214913.6703 | 962806.1705 | Riverine forest | 1206 |
| 160 | 214699.2037 | 963014.8442 | Riverine forest | 1620 |
| 161 | 214695.4709 | 963021.5532 | Riverine forest | 1302 |
| 162 | 214648.7971 | 963174.8361 | Riverine forest | 1203 |
| 163 | 214610.0879 | 963297.921 | Riverine forest | 1045 |
| 164 | 214657.8049 | 963713.5835 | Riverine forest | 1025 |
| 165 | 214543.893 | 963977.5868 | Riverine forest | 1036 |
| 166 | 214543.8986 | 963979.5071 | Riverine forest | 1089 |
| 167 | 214526.1065 | 964078.971 | Riverine forest | 1064 |
| 168 | 214526.1065 | 964211.2145 | Riverine forest | 1203 |
| 169 | 214538.675 | 964323.5537 | Riverine forest | 1230 |
| 170 | 214570.4067 | 964528.8631 | Riverine forest | 1254 |
| 171 | 214569.6763 | 964662.4392 | Riverine forest | 1263 |
| 173 | 214570.4067 | 964710.2017 | Riverine forest | 1630 |
| 174 | 214570.4067 | 964805.6851 | Riverine forest | 1480 |
| 175 | 214471.4244 | 964980.529 | Riverine forest | 1560 |
| 176 | 214467.1014 | 965004.2281 | Riverine forest | 1630 |
| 177 | 214452.3496 | 965081.5009 | Riverine forest | 1480 |
| 178 | 214453.1017 | 965126.468 | Riverine forest | 1203 |
| 179 | 214454.9194 | 965139.8853 | Riverine forest | 1420 |
| 180 | 214608.1458 | 965379.1708 | Riverine forest | 1500 |
| 181 | 214815.2886 | 965553.6743 | Riverine forest | 1503 |
| 182 | 214923.2197 | 965729.1697 | Riverine forest | 1523 |
| 183 | 214989.051 | 966278.7732 | Riverine forest | 1502 |
| 184 | 214856.7196 | 966464.6271 | Riverine forest | 1523 |
| 185 | 214615.3114 | 966668.3791 | Riverine forest | 1530 |
| 186 | 214437.3778 | 966819.3132 | Riverine forest | 1503 |
| 187 | 214287.0249 | 966948.4216 | Riverine forest | 1560 |
| 188 | 214039.0974 | 967076.2266 | Riverine forest | 1520 |
| 189 | 213859.6964 | 967108.6858 | Riverine forest | 1563 |
| 190 | 213836.016 | 967368.4599 | Riverine forest | 1200 |
| 191 | 213824.0733 | 967595.2544 | Riverine forest | 1201 |
| 192 | 213743.2095 | 967788.8934 | Riverine forest | 1209 |
| 193 | 213618.9016 | 967943.8692 | Riverine forest | 1208 |
| 194 | 213518.7466 | 968008.7178 | Riverine forest | 1206 |
| 195 | 213358.7665 | 968058.5999 | Riverine forest | 1263 |
| 196 | 213149.3784 | 968045.0226 | Riverine forest | 1268 |
| 197 | 213068.2846 | 968041.1956 | Riverine forest | 1274 |
| 198 | 213012.8637 | 968040.2267 | Riverine forest | 1248 |
| 199 | 212966.3768 | 968051.9399 | Riverine forest | 1256 |
| 200 | 212943.6082 | 968059.5977 | Riverine forest | 1286 |
| 201 | 212863.0659 | 968125.3323 | Riverine forest | 1289 |
| 202 | 212766.5927 | 968321.589 | Riverine forest | 1287 |
| 203 | 215284 | 961636 | Savanna grassland | 1274 |
| 204 | 215260 | 96163 | Savanna grassland | 1273 |
| 205 | 215210 | 96163 | Savanna grassland | 1271 |
| 206 | 215176 | 96163 | Savanna grassland | 1286 |
| 207 | 215152 | 96163 | Savanna grassland | 1295 |
| 208 | 215139 | 96163 | Savanna grassland | 1296 |
| 209 | 215132 | 96163 | Savanna grassland | 1294 |
| 210 | 215068 | 96163 | Savanna grassland | 1297 |
| 211 | 214823 | 96163 | Savanna grassland | 1299 |
| 212 | 214906 | 96163 | Savanna grassland | 1204 |
| 213 | 214992 | 96163 | Savanna grassland | 1203 |
| 214 | 215069 | 96163 | Savanna grassland | 1284 |
| 215 | 215144 | 96163 | Savanna grassland | 1265 |
| 216 | 215197 | 96163 | Savanna grassland | 1223 |
| 217 | 215290 | 96163 | Savanna grassland | 1211 |
| 218 | 215296 | 96163 | Savanna grassland | 1244 |
| 219 | 215276 | 96163 | Savanna grassland | 1245 |
| 220 | 215253 | 96163 | Savanna grassland | 1246 |
| 221 | 215313 | 96163 | Savanna grassland | 1256 |
| 222 | 215284 | 96163 | Savanna grassland | 1274 |
| 223 | 213409 | 968289 | Seasonally flooded grassland | 1274 |
| 224 | 213060 | 968498 | Seasonally flooded grassland | 1266 |
| 225 | 212984 | 968512 | Seasonally flooded grassland | 1261 |
| 226 | 212894 | 968527 | Seasonally flooded grassland | 1288 |
| 227 | 212794 | 968536 | Seasonally flooded grassland | 1292 |
| 228 | 212642 | 968561 | Seasonally flooded grassland | 1286 |
| 229 | 212620 | 968522 | Seasonally flooded grassland | 1282 |
| 230 | 212641 | 968445 | Seasonally flooded grassland | 1206 |
| 231 | 212701 | 968272 | Seasonally flooded grassland | 1254 |
| 232 | 212747 | 968187 | Seasonally flooded grassland | 1255 |
| 233 | 212868 | 968054 | Seasonally flooded grassland | 1245 |
| 234 | 212978 | 968021 | Seasonally flooded grassland | 1278 |
| 235 | 213182 | 968020 | Seasonally flooded grassland | 1263 |
| 236 | 213188 | 968024 | Seasonally flooded grassland | 1236 |
| 237 | 215542 | 960528 | Seasonally flooded grassland | 1230 |
| 238 | 215446 | 960546 | Seasonally flooded grassland | 1250 |
| 239 | 215316 | 960508 | Seasonally flooded grassland | 1245 |
| 240 | 215195 | 960461 | Seasonally flooded grassland | 1262 |
| 241 | 215065 | 960420 | Seasonally flooded grassland | 1263 |
| 242 | 215037 | 960391 | Seasonally flooded grassland | 1267 |
| 243 | 215043 | 960362 | Seasonally flooded grassland | 1289 |
| 244 | 215121 | 960342 | Seasonally flooded grassland | 1287 |
| 245 | 215169 | 960342 | Seasonally flooded grassland | 1256 |
| 246 | 215223 | 960327 | Seasonally flooded grassland | 1248 |
| 247 | 215319 | 960283 | Seasonally flooded grassland | 1236 |
| 248 | 215365 | 960269 | Seasonally flooded grassland | 1285 |
| 249 | 215388 | 960121 | Seasonally flooded grassland | 1245 |
| 250 | 215520 | 960178 | Seasonally flooded grassland | 1245 |
| 251 | 215590 | 960192 | Seasonally flooded grassland | 1268 |
| 252 | 215580 | 960354 | Seasonally flooded grassland | 1267 |
| 253 | 215542 | 960528 | Seasonally flooded grassland | 1264 |
| 254 | 218092.48 | 955183.054 | Riverine forest | 1349 |
| 255 | 216180.64 | 957435.512 | Riverine forest | 1386 |
| 256 | 222359.872 | 953199.067 | Riverine forest | 1315 |
| 257 | 216105.126 | 958973.479 | Riverine forest | 1309 |
| 258 | 215283 | 961815 | Riverine forest | 1350 |
| 259 | 213755.414 | 967456.114 | Riverine forest | 1050 |
| 260 | 218092.48 | 955183.054 | Natural forest | 1100 |
| 261 | 218019 | 956409 | Natural forest | 1200 |
| 262 | 216180.64 | 957435.512 | Riverine forest | 1120 |
| 263 | 222380.98 | 954083.57 | Natural forest | 1120 |
| 264 | 222359.872 | 953199.067 | Riverine forest | 1200 |
| 265 | 216105.126 | 958973.479 | Riverine forest | 1300 |
| 266 | 215337 | 962094 | Savanna grassland | 1315 |
| 267 | 213409 | 968289 | Seasonally flooded grass land | 1356 |
| 268 | 215542 | 960546 | Seasonally flooded grass land | 1358 |

**Table S3: Suitability classes results for each criteria/factors**

|  | Grazing suitability |  |  |
| --- | --- | --- | --- |
| no | Class name | Area(Ha) | % |
| 1 | Highly suitable | 5336.1 | 23.19 |
| 2 | Moderately suitable | 4257.33 | 18.49 |
| 3 | No suitable | 13419 | 58.31 |
|  | total | 23013 | 100 |
|  | Settlement |  |  |
| no | Class name | Area(Ha) | % |
| 1 | Highly disturbed | 10649.1 | 46.27 |
| 2 | Moderately disturbed | 7805.1 | 33.9 |
| 3 | No disturbed | 4559.9 | 19.8 |
|  | total | 23013 | 100 |
|  | Slope |  |  |
| no | Class name | Area(Ha) | % |
| 1 | Highly suitable | 1830.93 | 7.96 |
| 2 | Moderately suitable | 6057.69 | 26.32 |
| 3 | No suitable | 15125 | 65.72 |
|  | total | 23013 | 100 |
| no | water |  |  |
| 1 | Highly suitable | 7493.7 | 32.56 |
| 2 | Moderately suitable | 11226.4 | 48.78 |
| 3 | No suitable | 4293.2 | 18.66 |
|  | total | 23013 | 100 |


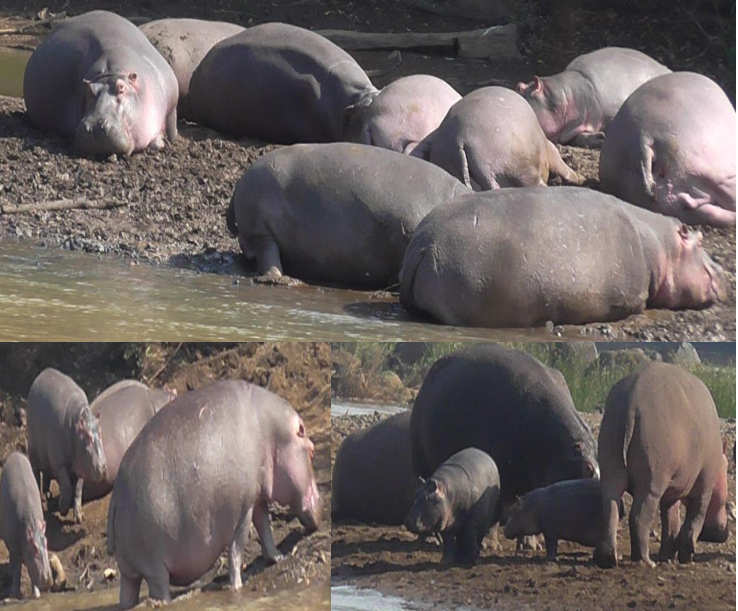


**Figure S1: Population size and herds of hippo in the study area**


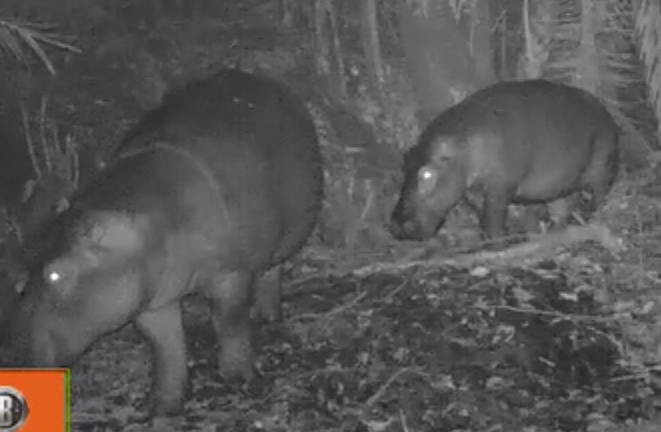


**Figure S2**: **Age structure of** **hippopotamus captured by camera trap in the study area**


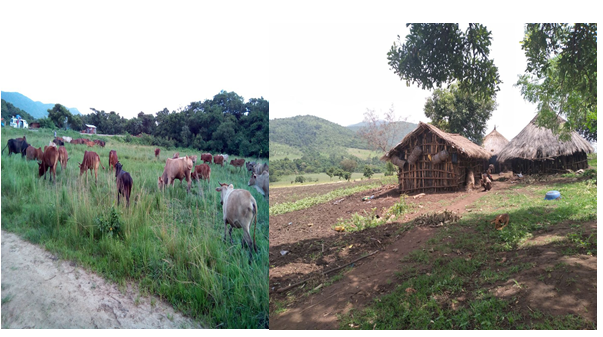


**Figure S3: Settlements and livestock disturbance on hippo habitat in the study area**


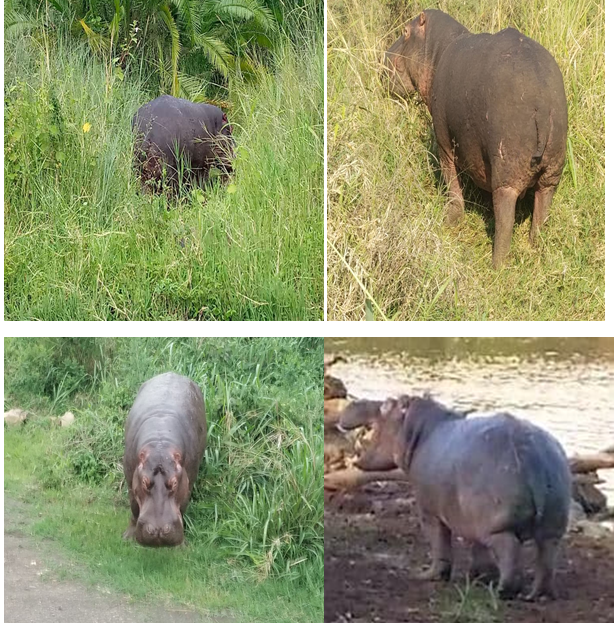


**Figure S4: Habitat preference of hippopotamus in study area during different season**
